# Fluoride adsorption on γ − Fe_2_O_3_ nanoparticles

**DOI:** 10.1186/s40201-015-0210-2

**Published:** 2015-07-24

**Authors:** Lakmal Jayarathna, Athula Bandara, W.J. Ng, Rohan Weerasooriya

**Affiliations:** Material Technology Section, Industrial Technology Institute, No 363, Bauddhaloka Mawatha, Colombo 07, Sri Lanka; Chemical and Environmental System Modeling group, Institute of Fundamental Studies, Hanthana Road, Kandy, Sri Lanka; Department of Chemistry, University of Peradeniya, Peradeniya, Sri Lanka; Nanyang Environment and Water Research Institute, Singapore, Singapore; Department of Soil Science, University of Peradeniya, Peradeniya, Sri Lanka

**Keywords:** γ-Fe_2_O_3_ nanoparticles, Fluoride, FTIR, Adsorption and removal, High efficiency, DFT, Molecular modeling, Gaussian 09

## Abstract

**Background:**

Fluoride contamination of groundwater, both anthropogenic and natural, is a major problem worldwide and hence its removal attracted much attention to have clean aquatic systems. In the present work, removal of fluoride ions from drinking water tested using synthesized γ-Fe_2_O_3_ nanoparticles.

**Methods:**

Nanoparticles were synthesized in co-precipitation method. The prepared particles were first characterized by X-ray diffraction (XRD) and Transmission Electron Microscope (TEM). Density functional theory (DFT) calculations on molecular cluster were used to model infrared (IR) vibrational frequencies and inter atomic distances.

**Results:**

The average size of the particles was around 5 nm initially and showed a aggregation upon exposure to the atmosphere for several hours giving average particle size of around 5–20 nm. Batch adsorption studies were performed for the adsorption of fluoride and the results revealed that γ-Fe_2_O_3_ nanoparticles posses high efficiency towards adsorption. A rapid adsorption occurred during the initial 15 min by removing about 95 ± 3 % and reached equilibrium thereafter. Fluoride adsorption was found to be dependent on the aqueous phase pH and the uptake was observed to be greater at lower pH. Fourier transform infrared spectroscopy (FT-IR) was used for the identification of functional groups responsible for the adsorption and revealed that the direct interaction between fluoride and the γ-Fe_2_O_3_ particles.

**Conclusions:**

The mechanism for fluoride removal was explained using the dehydoxylation pathway of the hydroxyl groups by the incoming fluoride ion. FT-IR data and other results from the ionic strength dependence strongly indicated that formation of inner-spherically bonded complexes. Molecular clusters were found to be good agreement with experimental observations. These results show direct chemical interaction with fluoride ions.

## Background

Fluorine is a naturally occurring element in minerals, geochemical deposits and natural water systems and that enters into food chains through either drinking water or eating plants and cereals [1]. Elevated concentrations of fluoride in soil and ground water arising from both natural and anthropogenic activities harm living beings around the world including Sri Lanka. Chemical weathering of some fluoride containing minerals leads to fluoride enrichment in soils and ground water. Discharge of fluoride from some industries, for example semiconductors, steel, etc. are among the main anthropogenic sources of fluoride pollution [2].

Removal of fluoride from water is one of the most important issues due to the effect on human health and environment. But as a necessary dilute element in human body fluoride in drinking water may be beneficial or detrimental depending on its concentration. Namely, dietary intake of fluoride with the concentration of 1 mg/L can prevent particularly skeletal and dental problems [3]. When the fluoride concentration is above this level, it leads to many bone diseases, mottling of teeth and lesions of the endocrine glands thyroid, liver and other organs. Owing to these adverse effects of fluoride, World Health Organization (WHO) accepted the drinking water with fluoride concentration of 1.5 mg/L [4]. In the literature, it was reported that many countries have regions where the water containing more than 1.5 mg/L of fluoride including north central province in Sri Lanka [5].

Recently, removal of fluoride from ground water and wastewater has been paid high attention in literature and different materials and methods have been tested. The mostly tested methods are adsorption [6–9], ion exchange [8], precipitation [10, 11], Donna dialysis [12], electrolysis [12] and nanofiltration [10, 12].

Among these methods, adsorption is the most widely used method for the removal of fluoride from water. Though these techniques have been extensively used in worldwide, but due to high cost, that methods are not suitable for field application [1].

Therefore, in recent years considerable attention has been devoted to the study of different types of low-cost and effective materials such as different clays, spent bleaching earths, alum sludge, red mud etc. in this approach, a large number of low-cost materials have been examined for the fluoride removal [5, 13–15]. However, to date, adsorbent of magnetic nanoparticles were reported very little, if any, to removal of fluoride from water solution where as magnetic nanoparticles adsorbent with excellent controllable properties can be developed for separation and removal ions from even very dilute aqueous solutions as the nanoparticles usually undergo modification of its geometric and electronic properties compared to bulk systems leading different pathways for the adsorption of molecules or atoms. Further, if the particles or the adsorbent possess magnetic properties then the main advantage is that the adsorbent can be easily separated using the external magnetic field and will be reused [16–20].

The most important solid surfaces for fluoride adsorption in water are the surfaces of Iron and Aluminum hydroxides, for example magnetite and gibbsite. In turn, adsorption of ions on hydroxide surfaces can affect the pH by influencing adsorption of protons. In the case of fluoride, adsorption of the negative ions enhances proton adsorption and tends to increase the pH. Although the amount of background electrolyte ions involved in this adsorption is generally minimal relative to the amount present in the solution. These effectively uncouple the adsorption of protons and fluoride and make the adsorption of fluoride at variable pH a multi-component process [21, 22].

The electronic and optical properties and the chemical reactivity of small clusters are completely different from the known properties of bulk or at extended surfaces. To overcome such difficulties, complex quantum mechanical models are required to predict the properties with particle size, and typically well defined conditions are needed to compare experiment results with theoretical predictions. The most important techniques in computational modeling are *ab-initio*, semi-empirical and molecular mechanics [23, 24].

Density functional theory (DFT) is a one of the newest approaches in computational modeling. In this method, the energy of the molecule and all of its derivative values depend on the determination of the wavefunction. Even though the wavefunction does not exist as a physically, observable property of an atom or a molecule, the mathematical determination of the wavefunction (within the atomic and molecular orbitals) is a good predictor of energy and other actual properties of the molecule [25].

This in turn was adapted by Kohn and Sham into a practical version of the density functional theory as follows,

$$ E\left[\rho \right]={T}_s\left[\rho \right]+\int \rho (r)V(r){d}^3r+\frac{1}{2}{\displaystyle \int \int \frac{\rho \left({r}_1\right)\rho \left({r}_2\right)}{r_{12}}}{d}^3{r}_1{d}^3{r}_2+{E}_{xc}\left[\rho \right] $$

Where, *T*_*s*_ is the kinetic energy of the non-interacting system; the second term is the nuclear attraction energy and the third is the classical coulomb self-energy; the last term is the *E*_*xc*_ energy. Each of these terms is a function of the function ρ, the electron density, which is itself a function of the three positional coordinates (x, y, and z) [26].

In this work, simple chemical method was used to synthesize magnetic iron oxide nanoparticles and employed to remove fluoride from solutions. Effects of pH and the background electrolyte were studied in the batch process. The FTIR spectroscopy was mainly used to characterize the systems in order to understand the adsorption mechanism of fluoride ions on the nanoparticles. Molecular modeling of the adsorbate-adsorbent interaction is very important to understand the surface complexation. Density functional theory, a type of *ab-initio* methods, applied to examine the atomistic and molecular level understanding of fluoride-γ-Fe_2_O_3_ interactions.

## Methods

All the chemicals used were in analytical grade.

Ferromagnetic iron oxide nanoparticles were synthesized by using modified co-precipitation of ferrous and ferric ions in alkaline medium [20]. Briefly an aqueous solution of Fe ions with molar ratio Fe(II)/Fe(III) = 0.5 was prepared by dissolving 3.25 g FeCl_3_ and FeCl_2_.4H_2_O powder in 60 mL of aqueous HCl acid (50 mL deionized water + 10 mL of 1 M HCl) solution. The resulting solution was added drop wise in to 100 mL of 1 M of NaOH solution under vigorous stirring. After all the Fe ions solution was added, the reaction mixture was stirred further to prevent coagulation of particles. Then, obtained colloidal solution was centrifuged at 2500 rpm, and precipitate was washed with deionized water with several times. Finally, precipitate was dried under normal atmospheric conditions.

### Characterization of iron oxide particles

Iron oxide particles were characterized by an X-ray diffraction (XRD) with an X-ray diffractometer equipped with a copper anode generating Cu K_α_ radiation (λ-1.5406 Ả). The surface structure, size and morphology were investigated by Transmission Electron Microscope (TEM). Fourier transform-infrared spectroscopy (Nicolet 6700 FT-IR) was also conducted on the particles. The surface area of iron oxide nanoparticles was estimated as 16.5 ± 2.5 m^2^/g according to Sears’ method, comparable with literature (20.40 m^2^/g) [27].

### Adsorption characteristics

Batch adsorption studies were conducted by contacting 10 g/L suspension of iron oxide particles with 20 mL of fluoride solution at varying concentrations (10–100 ppm) in polystyrene high-density tubes shaking for a 12 h, which had been shown in preliminary study to ensure equilibration to be reached. Temperature of adsorption test was ~25 ^0^C while the pH of the reaction mixture was adjusted in range of 2–12 using 0.1 M NaOH or 0.1 M HNO_3_. After shaking, the suspension was subjected for centrifugation and final fluoride ion concentration of the suspension was measured with a specific fluoride ion selective electrode (Orion 9409BN) by using an Orion EA960 auto-titrator. FTIR measurements in DRIFT mode were done on the residue solids obtained from each experiment in order to get insights into the mechanism of the fluoride adsorption on the iron oxide particles.

### Molecular modeling

Molecular modeling calculations were performed with “Gaussian 03” computer codes [28]. Models were built with GaussView tools. Molecular structures were determined by searching the potential energy surface for minima with respect to each atomic coordinates using density functional theory (DFT) calculations. Two ferrous atoms hydroxide octahedral cluster used as a basic of γ-Fe_2_O_3_ surface because this fragment is large enough to describe fluoride adsorption. These two octahedra connected by two OH bridge as they were in the crystal structure. Cluster configurations was performed using the DFT hybride B3LYP (Becke 3-term correlation functional; Lee, Yang, and Parr exchange function) function with 6-31G (*d, p*) basic set. Minimum energy structures were verified by calculating IR spectra for any imaginary frequencies (i.e., unstable vibrational modes). Calculated frequencies vs. experimental frequencies was plotted to examine the best-fit scale-factor “*m*”. It can be calculated as,

$$ \nu (scaled)=m.\nu \left(DFT/ basissets\right) $$

Where, *m* is the scale factor obtained from the slope of the plot, and *v* is the calculated frequencies for selected theory/basis set.

## Results and discussion

### Characterization of nanoparticles: XRD and TEM

**Fig. 1:**
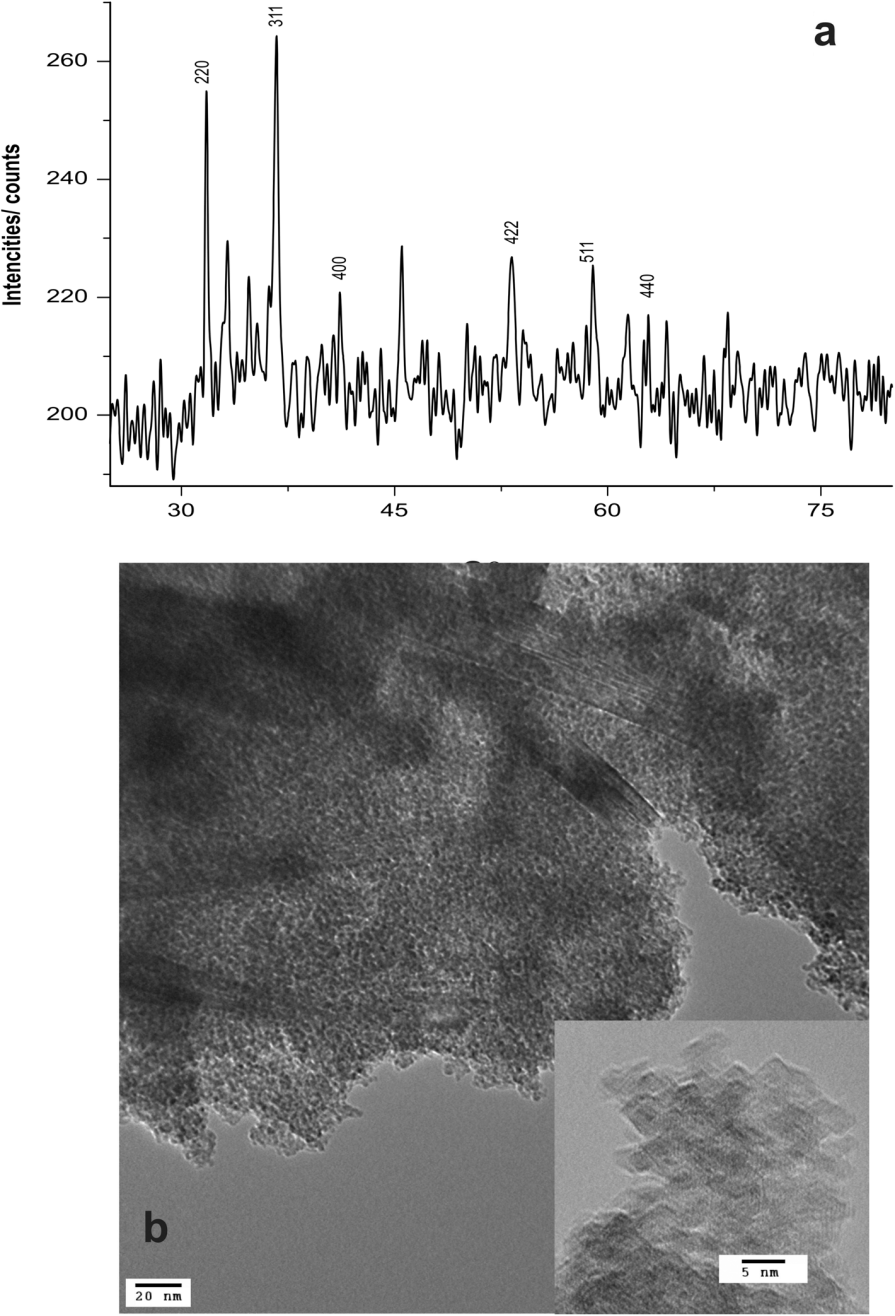
**a** XRD pattern of prepared γ-Fe_2_O_3_ particles. **b** TEM image of the particles: Particles tend to aggregate with the time of exposure to the atmosphere; initially the particles are around 5 - 20 nm in size

First, the crystal structure of synthesized nano particles was investigated by XRD using Cu K_α_ radiation. Figure 1(a) illustrates the XRD pattern, which matches well with that of γ-Fe_2_O_3_ [29]. Six characteristic peaks for γ-Fe_2_O_3_ (2θ =31.7^0^, 36.7^0^, 41.1^0^, 53.4^0^, 57.0^0^ and 62.6^0^) marked by their Miller indices (220), (311), (400), (422), (511) and (440), respectively, were observed [16]. As such the prepared particles showed high degree of crystalinity. Figure 1(b) shows the TEM image of the synthesized γ − Fe_2_O_3_ nanoparticles. As shown in Fig. 1(b), the powder consists of uniformly distributed spherical nanoparticles with particle size of 5–20 nm range, which is close to the calculated value (14.3 nm) from the XRD pattern. In bulk form, γ-Fe_2_O_3_ nanoparticles are spaniel cubic type and TEM figures illustrate high crystalline of the nanoparticles. However, particles are dry, it prefer to agglomerate with neighboring particles to reduce their surface charges and hence increasing the average size. Energy dispersive spectroscopy (EDS) also conformed that the ratio of Fe:O is in 2:3 ratio [30–33].

### Measurements on fluoride adsorption

**Fig. 2:**
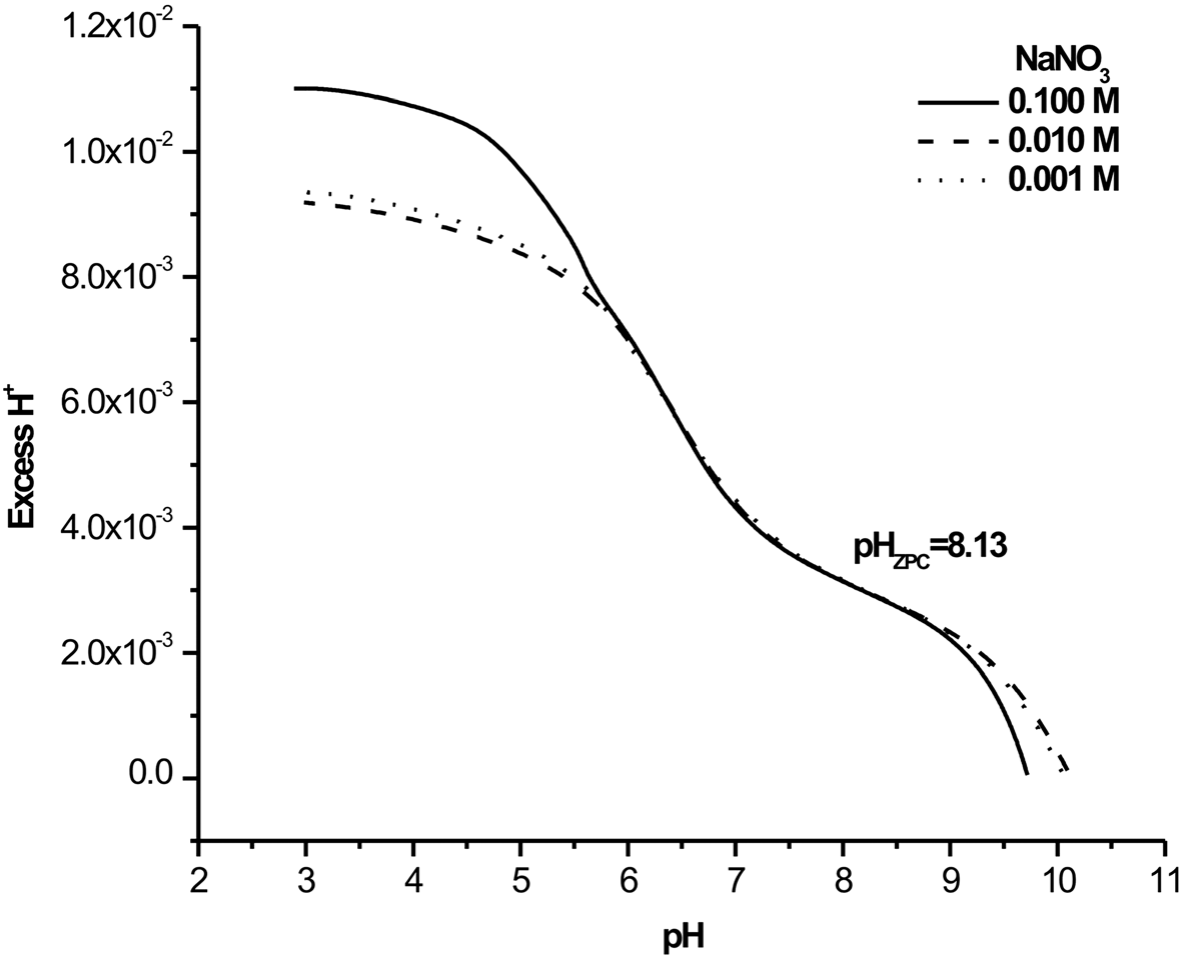
The graph showing zero-point-charge of the prepared γ-Fe_2_O_3_ particles obtained by surface titrations at different NaNO_3_ ionic strengths

The synthesized γ-Fe_2_O_3_ nanoparticles were first characterized by the surface titration in order to get an idea on the point of zero charge (ZPC) and the resulted titration curve is shown in Fig. 2. The observed value of zero point charge (pH_zpc_ = 8.13) suggests the presence of some weakly acidic groups on the surface of the adsorbent γ-Fe_2_O_3_ nanoparticles. According to literature data, the calculated pH_ZPC_$$ \left[=\left(\raisebox{1ex}{$1$}\!\left/ \!\raisebox{-1ex}{$2$}\right.\right)\left(p{K}_1+p{K}_2\right)\right] $$ is comparable with experimentally measured pH_ZPC_ (8.13) [34].

### Effect of pH of the solution on fluoride removal in different ionic strength

After characterizing the particles with ZPC the effect of pH on the adsorption of Fluoride was investigated. Fluoride adsorption by iron oxide was found to be strongly pH dependent. Adsorption amount decreased with increasing pH up to 4.5 and then remain more or less constant in the pH range of 6.0–10.0 and also that the adsorption remains almost constant regardless of ionic strength, but decreased slightly after pH >10.0. This may indicate the formation of inner-spherically bonded complexes [31, 35].

These results indicate that the adsorbent exhibits a commendable removal capacity in wide range of pH. At lower pH, below pH_zpc_, most of the surface sites are positively charged and attract negatively charged fluoride easily by electrostatic interaction. However, at very high pH, the removal capacity decreases due to the competition between hydroxide and fluoride ions in this medium [36].

It has also been observed that the removal of fluoride is very rapid in the first 15 min and then reaches a maximum. The percent fluoride removal after 15 min was found to be 95% at the pH 3.6 to 6. The change in the rate of removal might be due to the fact that initially all adsorbent sites were vacant and the solute concentration gradient is high. After 15 min, the fluoride uptake rate by adsorbent had been decreased due to the decrease in number of adsorbent sites. This removal percentage is remarkably higher than the systems reported earlier by using different types of clays [5]. This nature might be due to the modification of particles geometrically and electronically due to its nano size and also the high surface area of the small particles leads high affinity towards adsorption.

### FTIR measurements on the bare and fluoride adsorbed particles

**Fig. 3:**
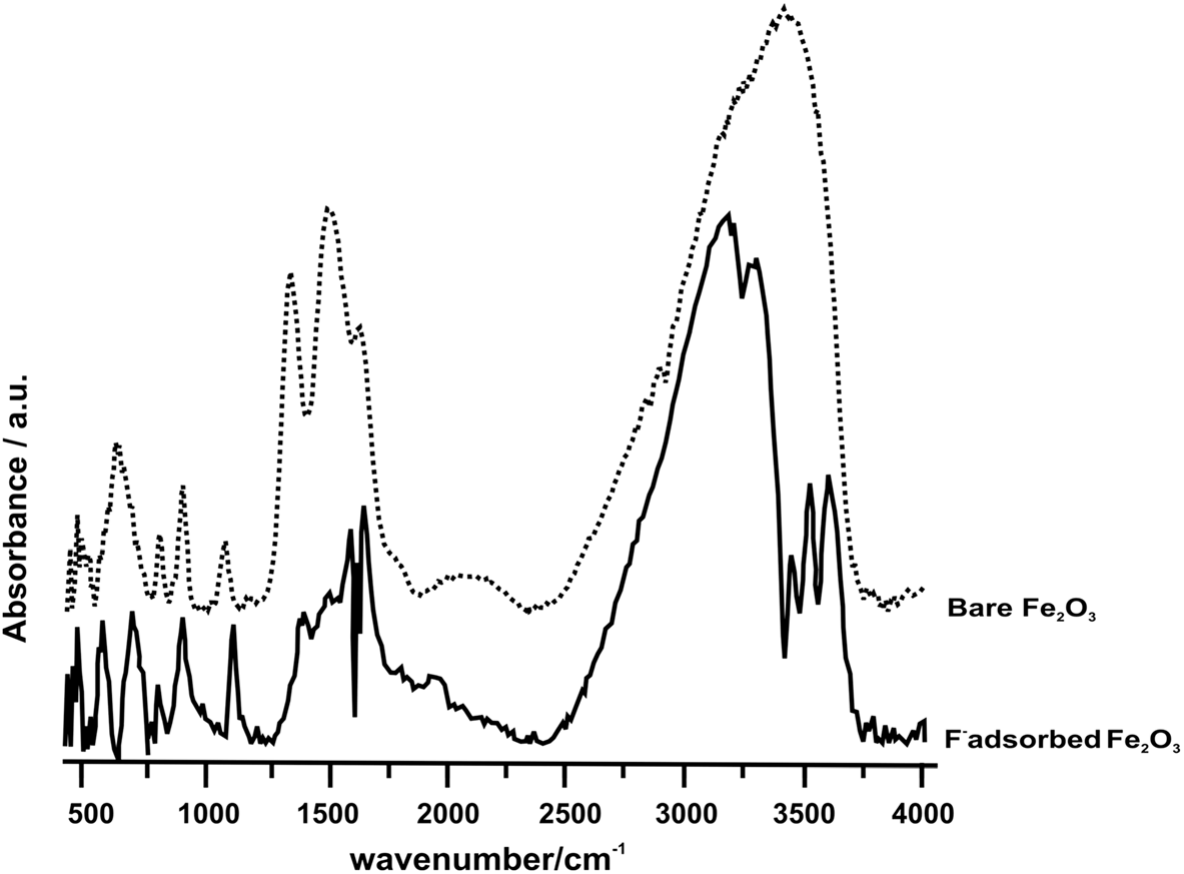
FTIR spectra of as prepared γ-Fe_2_O_3_ particles (dotted line) and the particles treated with saturation of fluoride solution at pH 6 (solid line). These two spectra compare the changes in the system upon adsorption of fluoride in the whole range of 500–4000 cm^-1^

FTIR measurements were carried out on the synthesized and fluoride adsorbed particles in order to characterize the systems with their nature of bonding. At first, the FTIR measurements were done on the bare particles to observe the existing functionalities and that the spectrum is shown by a dashed line in Fig. 3. IR absorption bands observed in the range 450–750 cm^-1^ are due to Fe-O bond vibrations and two sharp peaks at ~800 and ~900 cm^-1^ are due to the bending vibrations of O^…^Fe^…^O groups. The broad peak at around 3400 cm^-1^ is due to the hydrogen bonded OH as the surface adsorbed water is present on the particles and this further supported by the bands appearing around 1600 cm^-1^ [29, 32, 37].

Solid line in Fig. 3 shows the FTIR spectrum of fluoride adsorbed iron oxide nanoparticles. Adsorption bands at above 3000 cm^-1^ are well resolved now in comparison with the spectrum of bare-iron oxide. The IR absorption bands between 3300 and 3600 cm^-1^ are due to surface bound OH groups having their characteristic isolated nature. Indeed, a negative band at around 1620 cm^-1^ appeared and another band appeared at 1660 cm^-1^, revealing the presence of characteristic absorption bands for unbound surface water layer. The spectra shown in Fig. 3 are expanded to three different spectral regions in order to see the changes clearly and the following section deals with the major changes observed in the IR spectra upon the fluoride adsorption.

**Fig. 4:**
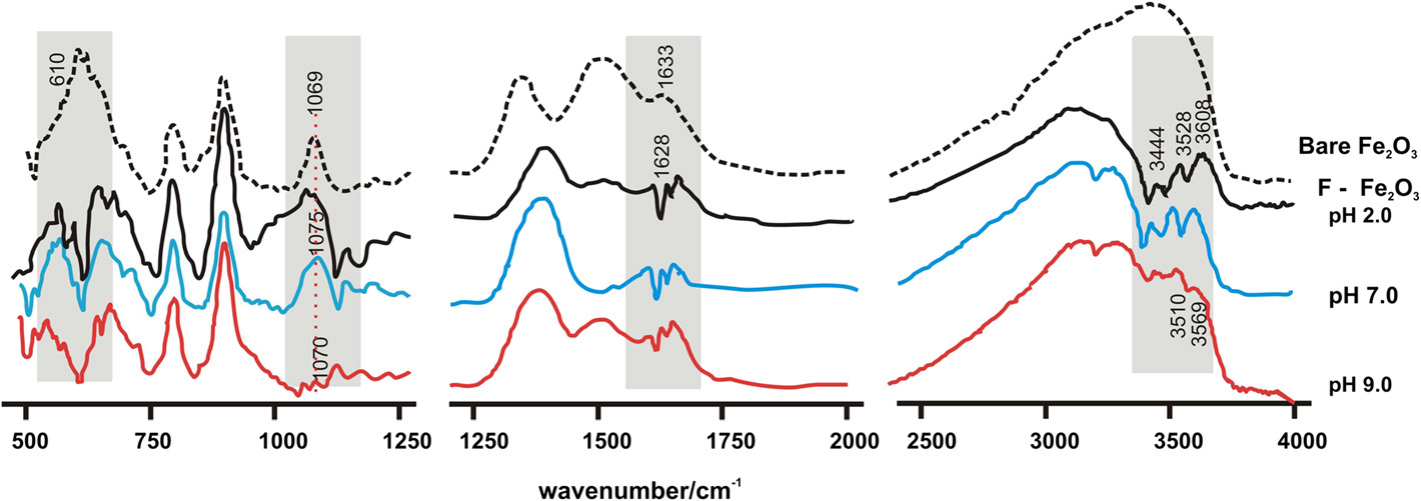
Series of FTIR spectra collected for the bare γ-Fe_2_O_3_ particles and those treated with fluoride under 0.001 M NaNO_3_. The wavenumber axis is broken into three different ranges of 500–1250, 1250–2000 and 2500–4000 cm^-1^ for the clarity to see the changes upon fluoride adsorption. The important areas are shaded to emphasis the major changes observed with marking the peak positions where the changes observed

According to the FT-IR spectral data, several significant changes can be observed in 500–1200 cm^-1^, 1200–2500 cm^-1^ and 2500–4000 cm^-1^ regions and these are shown in Fig. 4 measured at different pH with the background electrolyte of NaNO_3_ of 0.001 M. It has to be noted here that the spectra measured without background electrolyte for the fluoride adsorption showed similar pH dependent features except the remaining of some weak OH bands at around 3600 cm^-1^ at pH 9. Some negative absorption peaks appeared in 1200–2000 cm^−1^ region, while distinct features due to OH stretching modes appeared in the 2500–4000 cm^-1^ region upon the adsorption of fluoride on γ − Fe_2_O_3_ nanoparticles. Around 3000 to 3600 cm^-1^, five new peaks (at around 3182, 3295, 3442, 3528 and 3603 cm^-1^) observed after adsorbing fluoride. In the bare γ − Fe_2_O_3_ spectrum, a broad feature appeared in 2500 to 3700 cm^-1^ is due to hydrogen bonding behavior of surface adsorbed (physisorbed) water and surface > FeOH groups. However, after adsorbing fluoride, hydrogen-bonding strength was diminished and OH groups become much free from hydrogen bonding character and therefore, isolated surface OH vibrations are visible around 3600 cm^-1^. This nature is further evident by the splitting of the broad band around 600 cm^-1^ reflecting the clear Fe-O-H stretching vibrations free from hydrogen bonding and in addition, the appearance of negative absorption characteristics observed around 1630 cm^-1^ further supports the removal of hydrogen bonded OH sites. When considering the region 500 to 1200 cm^-1^, absorption band at 1069 cm^−1^ in bare γ − Fe_2_O_3_ shifted to higher wavenumbers upon the fluoride adsorption at neutral and acidic pH and the effect was minimum at pH 9. This effect decreased with increasing initial pH and the IR spectrum of the sample of pH 9 shows similar observation made with the bare γ − Fe_2_O_3_. Peaks at 1344, 1506 and 1634 cm^-1^are assign to bending modes of surface adsorbed water in bare iron oxide particles. The remaining characteristic absorption band at around 1660 cm^-1^ for fluoride adsorbed γ − Fe_2_O_3_ reveals the presence of some isolated OH groups even after the adsorption of fluoride ions. With the collection of all the features observed in the IR spectral ranges described above it is clearly evident that the fluoride adsorption occurs in inner-sphere mechanism as the observations clearly suggest that the direct bonding between surface > FeOH sites and incoming fluoride ion occur by replacing outer-spherically bonded water. This can also be proved by considering adsorption of fluoride in the presence of background electrolyte of NaNO_3_, where the amount of adsorption of fluoride does not depend on the ionic strength of the system. Further the nature of the particles as they have already modified electronically due to their nano dimensions may also influence the affinity to incoming ions with high efficiency. The changes observed in the IR spectra are negligible over the pH 9 and it is obvious that the particles start to dissolve when the point of zero charge is passed. By combining the pH dependence of IR data and other observations the following scheme is predicted for the adsorption of fluoride on the presently synthesized iron oxide particles [10, 18, 38].

**Fig. 5:**
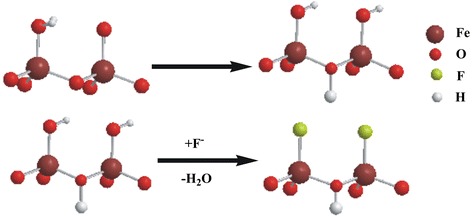
Proposed mechanism for the adsorption of fluoride on γ-Fe_2_O_3_ particles

In this scheme (Fig. 5), first, in the hydration step, surface adsorbed H_2_O molecules are formed on > FeOH sites of γ-Fe_2_O_3_ surface. Then, fluoride ions directly attached to the Fe atoms by replacing the OH groups bound to Fe by leaving lattice OH groups (>Fe_2_OH). As the size of F^-^ and OH^-^ are comparable and having the same charge, removal of OH^-^ by F^-^ may not be difficult in this event. Also the higher affinity of F^-^ than that of the OH^-^ towards Fe makes the replacement easier. As the Fe^…^F vibrations give rise IR bands in the region below 450 cm^-1^, that was not observed in the measured region, however, the appearance of negative IR bands around 1600 cm^-1^ and 3640 cm^-1^ clearly supports the above mechanism [34].

### Molecular modeling

**Fig. 6:**
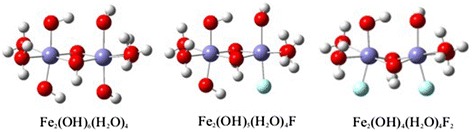
Fully optimized configurations of Fe_2_(OH)_6_(H_2_O)_4_, Fe_2_(OH)_5_(H_2_O)_4_F and Fe_2_(OH)_4_(H_2_O)_4_F_2_ clusters. Blue color in Fe, Red color in O, Off white color in H and Off green color in F

Optimized clusters of Fe_2_(OH)_6_(H_2_O)_4_, Fe_2_(OH)_5_(H_2_O)_4_F and Fe_2_(OH)_4_(H_2_O)_4_F_2_ were showed in Fig. 6. Fully optimized cluster for Fe_2_(OH)_6_(H_2_O)_4_ predicts average Fe-Fe and Fe-O bond length of 2.842 and 1.914 respectively. These bond distances are very much closer to literature data [39].

Table 1 shows optimized average bond distances for Fe_2_(OH)_6_(H_2_O)_4_, Fe_2_(OH)_5_(H_2_O)_4_F and Fe_2_(OH)_4_(H_2_O)_4_F_2_ clusters. Fluorine is highly electronegative and wishes to obtain additional electron density. It attempts to draw it from the other atoms which moves closer together in order to share the remaining electrons more easily as a result.

**Table 1:** Calculated bond parameters for Fe_2_(OH)_6_(H_2_O)_4_, Fe_2_(OH)_5_(H_2_O)_4_F and Fe_2_(OH)_4_(H_2_O)_4_F_2_ clusters

Computed bond distances Å			
Bond type	Fe_2_(OH)_6_(H_2_O)_4_	Fe_2_(OH)_5_(H_2_O)_4_F	Fe_2_(OH)_4_(H_2_O)_4_F_2_
Fe-Fe	2.842	2.843	2.489
Fe-O (Bridging)	1.914	1.914	1.869
Fe-O(H)	1.863	1.842	1.880
Fe-O(H_2_)	2.066	2.053	2.047
Fe-F		1.895	1.874

Cluster models were applied assuming the adsorption process is local phenomena. Such a model describing the surface adsorption sites can give important insights about the structure of the surface complexation. However, two ferrous atoms-hydroxide octahedral cluster (Fe_2_(OH)_6_(H_2_O)_4_) implemented as a basis of γ-Fe_2_O_3_ because this fragment is large enough to describe the fluoride adsorption and to avoid any complex time consuming calculation steps.

**Fig. 7:**
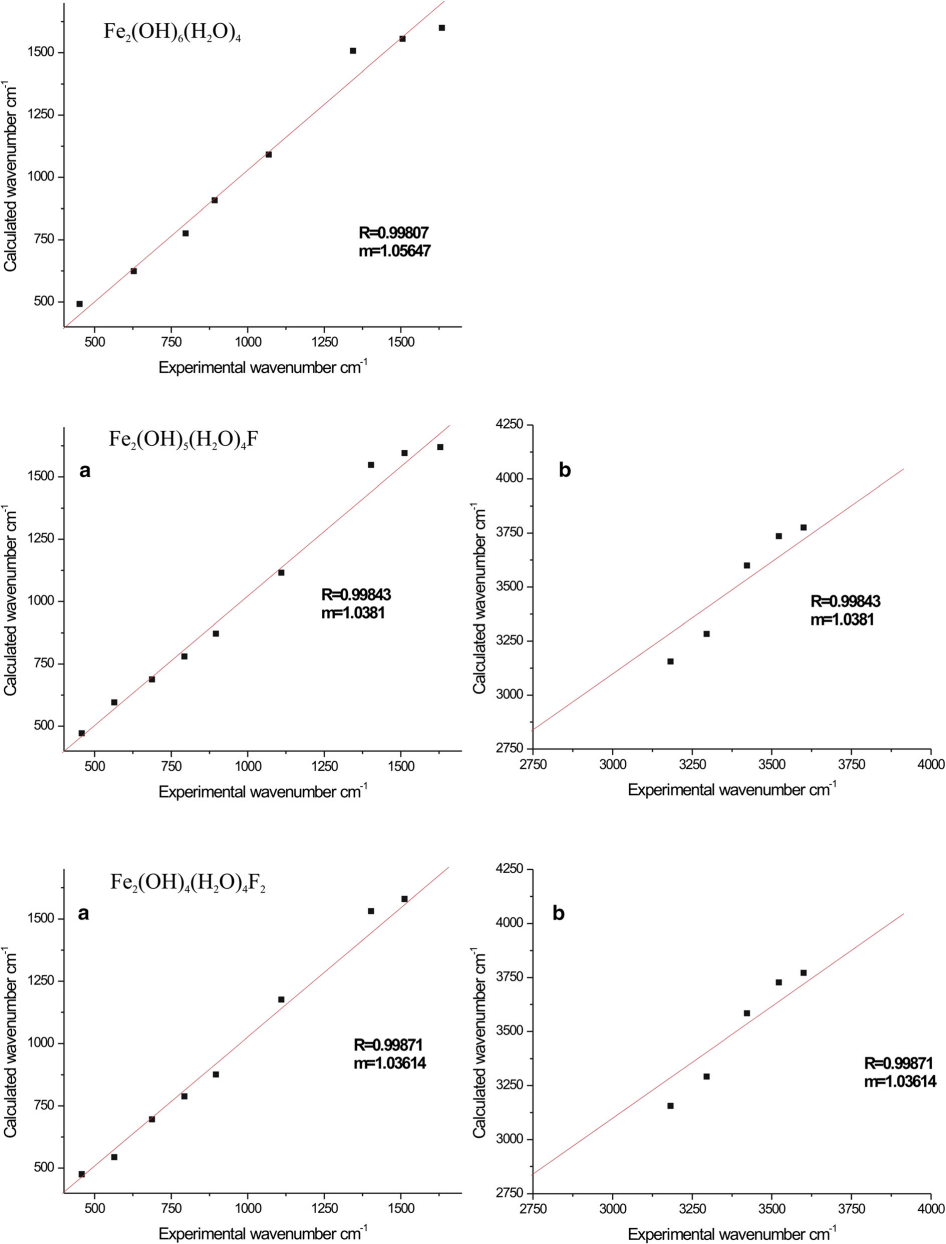
Frequency correlation of experimental and modeled modes of Fe_2_(OH)_6_(H_2_O)_4_, Fe_2_(OH)_5_(H_2_O)_4_F and Fe_2_(OH)_4_(H_2_O)_4_F_2_ clusters

Comparison between calculated and experimental vibration spectra of Fe_2_(OH)_6_(H_2_O)_4_, Fe_2_(OH)_5_(H_2_O)_4_F and Fe_2_(OH)_4_(H_2_O)_4_F_2_ clusters were shown in Fig. 7. Computed frequencies of the DFT calculation are closely related to the experimental FT-IR vibrations. Calculated OH bending frequencies of iron oxide cluster (Fe_2_(OH)_6_(H_2_O)_4_) in good agreement with the experimental observation bellow 1000 cm^-1^. However, Oh stretching frequencies is different due to H-bonding effects. The argument is noteworthy because a significant error is expected due to the fact that harmonic frequencies are calculated whereas anharmonic frequencies are observed. Generally, for more accuracy the scale factor (m) should be closer to one. In some cases, scale factor are actually found to be slightly greater than or lower than 1.0. However, in this calculations scale factor very much closer to one [25, 40, 41].

According to the calculated IR spectra of the Fe_2_(OH)_6_(H_2_O)_4_, Fe_2_(OH)_5_(H_2_O)_4_F and Fe_2_(OH)_4_(H_2_O)_4_F_2_ clusters, spectra show higher wavenumber shifting for Fe_2_(OH)_5_(H_2_O)_4_F and Fe_2_(OH)_4_(H_2_O)_4_F_2_ clusters. It is good indication for decreases of hydrogen bond strength for fluoride adsorbed clusters.

## Conclusion

Magnetic γ − Fe_2_O_3_ nanoparticles were synthesized by the co-precipitation method and this work confirmed that magnetic γ − Fe_2_O_3_ nanoparticles possess remarkably high efficiency for the removal of fluoride from drinking water and wastewater. The removal of fluoride capacity was 3.65 mg/g and it is strongly depended on initial pH of solution and the removal level is high as 95% of the removal occurs in acidic to neutral pH. FTIR measurements indicated the formation of inner-spherically bonded complexes and the removal of outer-sphere water molecules paved the way to appear isolated OH groups in the IR spectra upon the adsorption of fluoride and that the data helped us to predict the mechanism for the adsorption. Computational chemistry is very useful tool for studying surface reactions when used in combination with a variety of experimental techniques. Density Functional Theory (DFT) can reproduce the structure and vibration frequencies of bulk γ-Fe_2_O_3_ and these methods were also applied to predict the surface structure.
